# Protective effect of *Bletilla ochracea* Schltr. against acetogenic gastric ulcer in rats based on non-targeted metabolomics

**DOI:** 10.3389/fmed.2024.1447566

**Published:** 2024-11-28

**Authors:** Rongze Fang, Qi Zeng, Xiusheng Tang

**Affiliations:** ^1^School of Pharmacy, Guizhou University of Traditional Chinese Medicine, Guiyang, Guizhou, China; ^2^Acupuncture Rehabilitation Department, Cengong Hospital of Traditional Chinese Medicine, Kaili, Guizhou, China; ^3^Pharmacy Department, The First Affiliated Hospital of Guizhou University of Traditional Chinese Medicine, Guiyang, Guizhou, China

**Keywords:** gastric ulcer, *Bletilla ochracea* Schltr., non-targeted metabolomics, metabolic pathway, protective effect

## Abstract

**Background:**

Gastric ulcer (GU), a globally prevalent disease, represents a significant burden to human health. *Bletilla ochracea* Schltr. (BOS), an herbal medicine, shows promising therapeutic potential in the treatment of chronic GU.

**Methods:**

This study utilized a rat model of chronic gastric ulceration induced by acetic acid to evaluate the protective effects of *Bletilla ochracea* Schltr. (BOS) on gastric tissue through the analysis of gross morphological and histopathological changes. Non-targeted metabolomic techniques were employed to identify differential metabolites, followed by the use of metabolic analysis software to enrich the pathways associated with these metabolites, thereby revealing the potential mechanisms underlying the anti-gastric ulcer effects of BOS.

**Results:**

The results suggest that the primary mechanism underlying BOS regulation of GU involves modulation of endogenous metabolites, including dimethylglycine, l-2,4-diaminobutyric acid, uridine propionic acid and l-asparagine. These diverse metabolites may have anti-inflammatory, antioxidant and reparative properties. In addition, KEGG enrichment analysis indicated potential anti-GU effects of BOS through diverse pathways such as energy metabolism, immune metabolism and amino acid metabolism.

**Conclusion:**

The study demonstrates BOS protective effects on GU in rats, potentially through modulating key metabolites and pathways, highlighting its therapeutic potential and warranting further investigation for clinical applications.

## 1 Introduction

Gastric ulcer (GU) is a globally prevalent stomach pathology characterized by substantial necrotic tissue damage, bleeding, and perforation affecting both the mucosal surface and muscularis mucosa, posing a considerable threat to the patient’s overall health and well-being (1). The etiology of GU is multifaceted, often encompassing a complex interplay of various factors such as gastric mucosal injury, hypersecretion of gastric acid, and *Helicobacter pylori* infection. The primary manifestations of GU encompass upper abdominal pain, heartburn, and acid reflux (2–4). Despite significant advancements in modern healthcare, managing gastrointestinal ulcers remains challenging, particularly due to potential functional limitations associated with prolonged use of proton pump inhibitors (PPIs), histamine H2 receptor blockers, and antacids. These limitations can lead to sexual mucosal alterations, disruptions in intestinal flora balance, and other adverse side effects (5, 6). Consequently, there is a dire need to investigate alternative and safer therapeutic strategies aimed at alleviating the discomfort and enhancing the quality of life for individuals afflicted with GU.

In Traditional Chinese Medicine, *Bletilla ochracea* Schltr. (BOS) plays an important role in the treatment of gastric disorders. BOS, an ancient medicinal herb, exhibits a wide range of biological activities, including anti-inflammatory, antioxidant and antimicrobial properties, making it a popular choice for the treatment of gastrointestinal disorders, particularly GU (7). Previous research has demonstrated the ability of BOS to prevent and alleviate gastric inflammation, while also helping to protect the gastric mucosa from damage (8, 9). However, further extensive research is required to elucidate the intricate mechanisms, safety profile and therapeutic efficacy of BOS and ultimately guide its potential clinical use. These efforts will contribute to an accurate understanding of the role of BOS in the treatment of GU and provide a more robust scientific basis for its expanded use in traditional Chinese medicine.

In this study, non-targeted metabolomics techniques were used to investigate the protective effect of BOS on acetic acid-induced GU in rats. Through in-depth analysis of the changes in the metabolic spectrum *in vivo*, the key metabolic pathways closely related to treatment efficacy were revealed. The systematic evaluation of BOS provides a scientific basis for its application in the treatment of GU disease (10). In addition, this study provides a detailed insight into the metabolic effects of BOS, which should open up new perspectives for future research and contribute to the development of more effective strategies for the treatment of GU. This study is expected to enhance our understanding of BOS and its mechanism for the treatment of GU and at the same time provide strong support for promoting the modernization of traditional Chinese medicine research and application.

## 2 Materials and methods

### 2.1 Reagents

LC–MS grade acetonitrile (ACN) was purchased from Dikma Technologies (51 Massier Lane, United States). Formic acid was provided by TCI (Shanghai, China). Ammonium formate was supplied by Sigma-Aldrich (Shanghai, China). Watsons in Guangdong, China supplied the ultrapure water. A microporous membrane with a pore size of 0.22 μm was purchased from Tianjin Jinteng Experiment Equipment Co., Ltd. in Tianjin, China. Acetic acid was purchased from Tianjin Comeo Chemical Reagent Co., Ltd. with batch number 20230110. Ranitidine Capsules, purchased from Suzhou Hongsen Pharmaceutical Co, Ltd., Lot No: 306220503.

### 2.2 Laboratory equipment

A high speed centrifuge was purchased from Hunan Xiangyi Experiment Equipment Co., Ltd. (Hunan, China). The centrifugal vacuum evaporator was obtained from Beijing Jiaimli Technology Co, Ltd. (Beijing, China). The vortex mixer was supplied by Sinopharm Chemical Reagent Co, Ltd. in Shanghai, China.

### 2.3 Sample source and handling

#### 2.3.1 Sample processing

BOS, obtained from Baihua Town, Cuiping District, Yibin City, Sichuan Province, was identified as the dried tuber of the orchid plant BOS by Professor Wang Xiangpei of Guizhou University for Nationalities. According to the 2003 edition of “Guizhou Province Chinese Medicinal and Ethnic Medicinal Materials Quality Standards,” the recommended dosage of BOS is 6–15 g. The group’s previous research (9) found that the dosage of 15 g of BOS was the best for the pre-test, and there was no stress reaction and death after the rats were given the drug by gavage, so the dosage of 15 g of BOS was selected for this study, referring to the “Methodology of Pharmacological Research of Traditional Chinese Medicines” (11) for the equivalent dosage conversion method between human and experimental animals, the equivalent dose for rats is 1.35 g per kilogram, with a higher dose of 2.7 g per kilogram. To prepare the extract, an appropriate amount of BOS is mixed with eight times its volume of water and boiled three times. Before the first boiling, the mixture is soaked in water for 30 min and then boiled vigorously until it comes to the boil. The heat is then reduced to maintain a gentle boil for 30 min. The filtrate from each boil is combined, dried and concentrated to produce the liquid extract, which is stored in a refrigerator for future use. When used, the extract should be prepared on the basis of a crude drug content of 2.7 g per kilogram.

#### 2.3.2 Preparation of acetic acid solution

Measure precisely 30 mL of acetic acid and dilute it with distilled water to achieve a final volume of 200 mL, yielding a solution with a volume fraction of 15%. Ensure that this solution is prepared freshly and consumed on a daily basis.

### 2.4 Animal experiment

Male Sprague–Dawley rats weighing between 180 and 220 g, all SPF grade, were used in this study and were obtained from the Animal Institute of Guizhou University of Traditional Chinese Medicine. The license number for the production of these laboratory animals is SYXK (Guizhou) 2021–0005. To ensure their well-being, the animals were housed in an environment maintained at a temperature of (25 ± 2)°C with a relative humidity of 40–60%. The rats were fasted for 12 h before the start of the experiment, but were allowed to drink water freely. The research protocol was approved by the Ethics Committee of Guizhou University of Traditional Chinese Medicine, the approval number is 20230004.

### 2.5 Animal simulation and medication administration

The rats were given intelligent diets for a period of 1 week. Using the random number table technique, the animals were divided into four different groups, each consisting of six rats: the control group, the model group, the Ranitidine group and the BOS group. Except for the control group, which did not undergo any intervention, all other animals underwent acetic acid treatment to establish a GU model. Specifically (12, 13), 1 mL of dose of 15% acetic acid was administered daily for four consecutive days. After completion of the modeling, the BOS group received an intragastric dose of 2.7 g/kg, the Ranitidine group (14) received an intragastric dose of 0.3 g/kg and 10 mL/kg, which was determined based on previous experimental results. Both the control and model groups received an equal volume of distilled water daily for 10 consecutive days.

### 2.6 Sample collection and analysis

On the ninth day after dosing, the rats were fasted for 12 h while maintaining regular access to water. One hour after the last treatment, the rats were sedated by intraperitoneal injection of 20% urethane (6 mL/kg). Blood samples were then taken from the abdominal aorta and gastric tissue was harvested. Gastric tissue was rinsed with normal saline and stored at −80°C. Plasma was centrifuged at 3,500 rpm for 15 min and the supernatant was collected for subsequent analysis.

### 2.7 Histopathological changes in the stomach

Gastric tissues were immersed in 10% formalin for more than 48 h, dehydrated in a graded series of ethanol and finally embedded in paraffin. The paraffin-embedded stomach block was securely fixed to the specimen holder to ensure complete exposure of the stomach section. Subsequently, 4 μm thick sections were cut and mounted with neutral gum. Microscopic examination was performed to observe tissue morphology and structural changes in the stomach.

### 2.8 Metabolomic analysis of rat plasma

#### 2.8.1 Metabolite extraction

Thaw the test plasma under controlled conditions at 4°C. After thawing, mix the samples vigorously by vortexing for 1 min to ensure homogeneity. Carefully transfer the prescribed 300 μL of sample to a 2 mL centrifuge tube. Add 400 μL of methanol, shake vigorously for 1 min and centrifuge at 12,000 rpm for 10 min at 4°C. Collect the supernatant, i.e., the liquid above the sediment, and transfer it to a fresh 2 mL centrifuge tube. Evaporate the supernatant to condense it. Carefully add 150 μL of a 2-chlorophenylalanine solution prepared at a concentration of 4 ppm in a mixture of 80% methanol and water to the sample. Pass the supernatant through a 0.22 μm filter to remove any particles. Transfer the filtered liquid to a detection vial for liquid chromatography-mass spectrometry (LC–MS) analysis (15).

#### 2.8.2 Chromatographic conditions

The Waters ACQUITY ultra-high performance liquid chromatography system was used with an ACQUITY UPLC^®^ HSS T3 column (2.1 × 150 mm, 1.8 μm) from Waters, Milford, MA, United States, at a flow rate of 0.25 mL/min. The flow rate, column temperature and injection volume were all set at 40°C and 2 μL, respectively. For chromatography in positive ion mode, the mobile phase contains 0.1% formic acid-acetonitrile (B1) and 0.1% formic acid-water (A1). This gradient elution method starts with 2% B1 from 0 to 1 min, increases to 50% B1 from 1 to 9 min, further increases to 98% B1 from 9 to 12 min, maintains 98% B1 from 12 to 13.5 min, decreases back to 2% B1 from 13.5 to 14 min and finally returns to 2% B1 from 14 to 20 min. Acetonitrile (B2) and 5 mM ammonium formate in water (A2) form the mobile phase for chromatography when operating in negative ion mode. The gradient elution program is as follows: from 0 to 1 min, the B2 concentration is 2%; from 1 to 9 min, the B2 concentration increases from 2 to 50%; from 9 to 12 min, the B2 concentration increases from 50 to 98%; from 12 to 13.5 min, the B2 concentration is 98%; from 13.5 to 14 min, the B2 concentration decreases from 98 to 2%; and from 14 to 17 min, the B2 concentration returns to 2% (16).

#### 2.8.3 Mass spectrometry conditions

Information was collected using a Thermo Q Exactive mass spectrometer detector from Thermo Fisher Scientific in the USA, together with an electrospray ion source (ESI) operating in both positive and negative ion modes. The positive ion spray voltage was set at 3.50 kV and the negative ion spray voltage at 2.50 kV, with a sheath gas pressure of 30 arb and an auxiliary gas pressure of 10 arb. The capillary temperature is set at 325 degrees Celsius and a first comprehensive scan is performed with a resolution of 70,000. The primary ion scan range covers m/z 100 to 1,000 using HCD for secondary fragmentation with a collision energy of 30 electron volts. The resolution for the second stage is 17,500, fragmenting the top 10 ions and implementing dynamic exclusion to eliminate irrelevant MS/MS data (17).

### 2.9 Data processing analysis

The MS Convert tool within the Via Proteowizard software (v3.0.8789) (18) is used to convert the original offline mass spectrum files into the mz XML file format. The R XCMS software package (19) was used to identify, filter and align peaks to generate a quantitative inventory of compounds. Specified parameters included bw = 2, ppm = 15, peakwidth = c (5, 20), mzwid = 0.015, m/zdiff = 0.01 and method = “centWave.” Public databases such as HMDB (21), massbank (22), LipidMaps (23), mzcloud (24), KEGG (25) and custom compound libraries were used for compound identification with parameters set to ppm < 30 ppm. The LOESS (26) signal correction method using QC samples corrects the data and removes systematic errors. Compounds with an RSD greater than 30% in the QC samples are excluded during the quality control process. Ropls (27) software was used to perform Principal Component Analysis (PCA), Partial Least Squares Discriminant Analysis (PLS-DA) and Orthogonal Partial Least Squares Discriminant Analysis (OPLS-DA) to reduce the dimensions of the sample data (*n* = 6), and various plots, including score plots, loading plots and S-plot plots, were generated to illustrate the variation in metabolite composition between samples. The permutation test technique was used to assess the model for overfitting. R2X and R2Y indicate the proportion of variance explained by the model for the X and Y matrices, while Q2 assesses the predictive performance of the model. The closer their values are to 1, the better the fit of the model and the more accurately the training set samples can be classified in their original assignment. Calculate the *p*-value by performing statistical tests, assess the Variable Importance of Projection (VIP) using the OPLS-DA dimensionality reduction technique, and evaluate the fold change to understand the impact and explanatory power of each metabolite component on sample classification and discrimination. Furthermore, these criteria were used as indicators to screen for metabolites. Metabolite compounds were considered statistically significant if they had a *p-*value below 0.05 and a VIP value greater than 1.

### 2.10 Pathway analysis

Functional pathway enrichment and topology analysis of the identified differential metabolites was performed using the MetaboAnalyst (28) software package. Differential metabolite and pathway maps were explored by using the KEGG Mapper visualization tool to navigate through the enriched pathways.

## 3 Results

### 3.1 Amelioration of apparent gastric mucosal damage in GU rats

**Figure 1:**
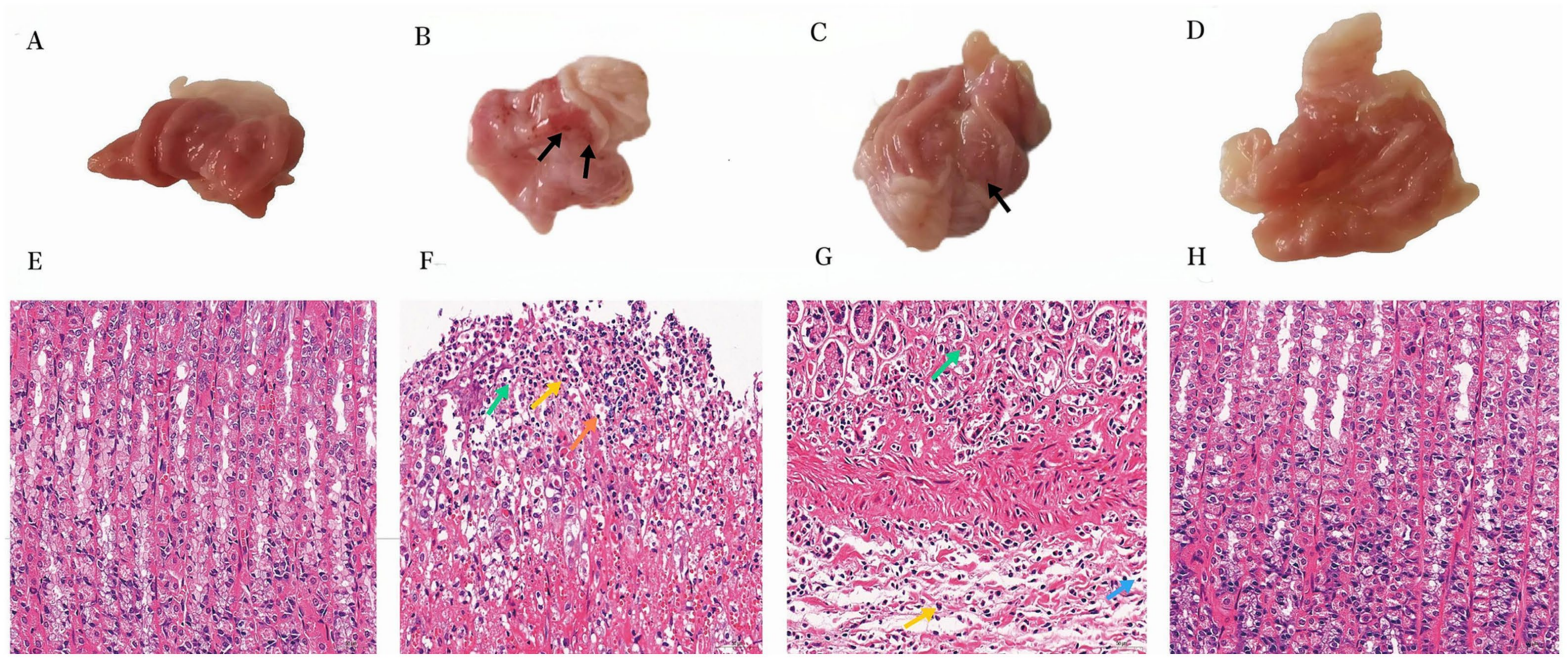
Apparent analysis and pathological analysis (400×) of gastric tissue in rats. **(A)** Gastric tissue from the control group. **(B)** Anhydrous ethanol induced GU (Model group). **(C)** Ranitidine −0.3 g/kg acetic acid-induced GU rats. **(D)** BOS −2.7 g/kg for treatment of acetic acid-induced GU rats. **(E)** Gastric histological section of control group. **(F)** Pathological section of gastric tissue in model group. **(G)** Histopathologic section of gastric tissue induced by Ranitidine −0.3 g/kg treatment in GU rats. **(H)** Pathological section of gastric tissue induced by acetic acid in GU rats treated with BOS −2.7 g/kg. Necrotic Cell Debris(Green); Lymphocytes (Orange/Blue); Neutrophils (Yellow).

One hour after the administration of the final dose, a thorough examination of the rats’ stomachs was conducted to assess the status of their gastrointestinal tract. In the control group, the gastric mucosal surface appeared smooth and pink, exhibiting a substantial quantity of mucus within the stomach cavity. Additionally, numerous mucosal folds were observed, and there were no indications of hemorrhage (Figure 1A). In contrast, the rats belonging to the model group exhibited extensive damage to their gastric lining, characterized by the presence of dark ulcers and visible tissue adhesion without the need for magnification (Figure 1B). Gastric mucosal bleeding in Ranitidine group was significantly less than that in model group (Figure 1C). Notably, in the BOS group, no apparent bleeding sites were discernible within the gastric mucosa of the rats. Moreover, their observable gastric structure closely resembled that of the control group, as depicted in Figure 1D.

### 3.2 Amelioration of pathological damage to the gastric mucosa in GU rats

In the control group, the gastric tissue of rats exhibited structurally intact layers of the mucous membrane, submucous membrane, muscular layer, and plasma membrane. The mucosal layer was covered by a single layer of columnar epithelium, with tubular gastric glands visible in the lamina propria. Cells were neatly organized, and blood vessels and nerves were present in the loose connective tissue of the submucosal layer. No apparent abnormalities were observed in the mesenchyme, muscularis propria, or tunica albuginea. In contrast, the model group demonstrated edema in the submucosal layer of the rat stomach, with a widened interval between the mucosal and muscular layers. Small amounts of hemorrhage and erythrocyte aggregation were also observed. Furthermore, inflammatory cell infiltration was evident in the necrotic region, primarily consisting of lymphocytes with round, deeply stained nuclei, and neutrophils with rod-shaped or lobulated nuclei. Edema and inflammatory cell infiltration in the submucosal layer of the gastric mucosa were improved in rats in the ranitidine group. In the BOS group, the stomach tissues of rats maintained structural integrity in all layers, including the mucosal, submucosal, muscular, and plasma membrane. A single layer of columnar epithelium covered the mucosal layer, with tubular gastric glands present in the lamina propria. Cells were neatly arranged, with mural cells primarily located on the superficial surface of the gastric glands. Main cells were predominantly found at the bottom of the glands, exhibiting cone or column shapes with basophilic cytoplasmic bottoms. Blood vessels and nerves were observed within the loose connective tissue of the submucosa, and no apparent abnormalities were detected in the mesenchyme, muscularis propria, or tunica albuginea (Figures 1E–H).

### 3.3 Metabolic profile results of rat plasma samples

**Figure 2:**
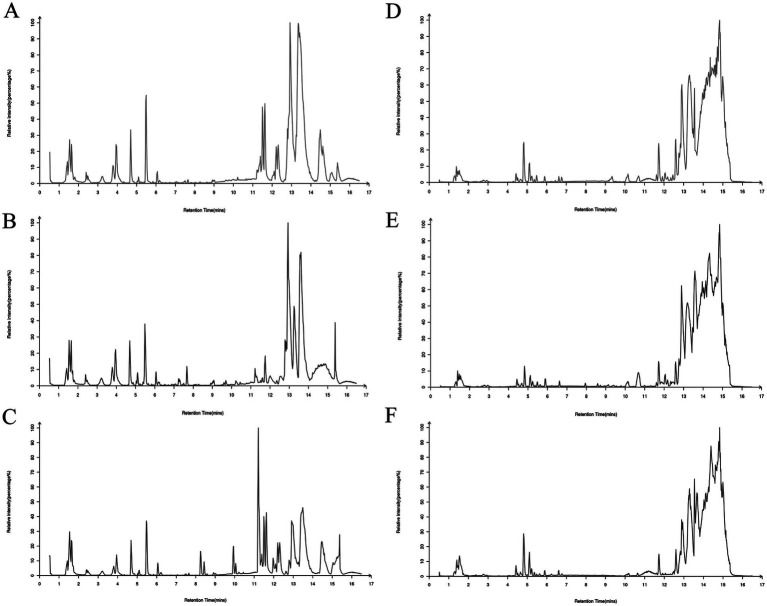
Total ion chromatogram (TIC) of rat plasma samples in both positive and negative ion modes. **(A)** Positive ion of control rats; **(B)** positive ion of model group rats; **(C)** positive ion of BOS group rats; **(D)** negative ion of control group rats; **(E)** negative ion of model group rats; **(F)** negative ion of BOS group rats.

A comprehensive analysis of rat plasma samples was performed using advanced UHPLC-Q-Exactive MS technology. This analysis was designed to separate the individual components and collect accurate data, ultimately resulting in the generation of total ion chromatograms (TICs) for the control, model and BOS groups in both positive and negative ion modes. These TICs are shown graphically in Figure 2. MS-DIAL software was used to extract the metabolite ion peaks. The results of this analysis showed the detection of 14,754 ion peaks in positive ion mode and 13,482 in negative ion mode, indicating significant variations in metabolite content between the different plasma sample groups.

### 3.4 PLS-DA analysis

**Figure 3:**
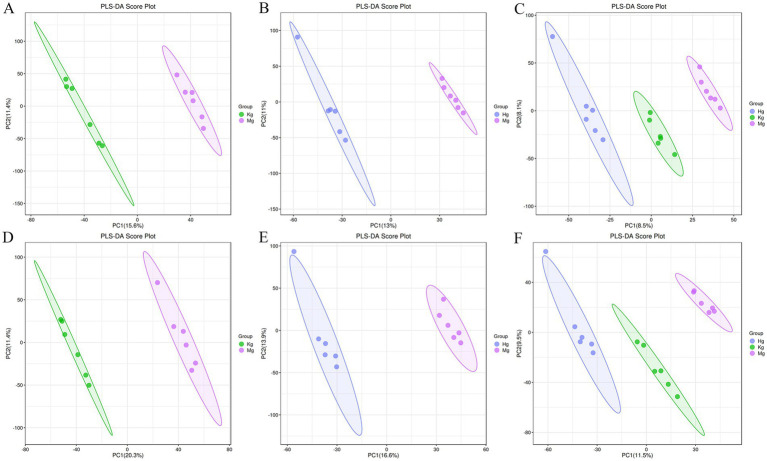
PLS-DA scores of positive and negative ion patterns in rat plasma samples. Kg represents the control group, Mg represents the model group, and Hg represents the BOS group. **(A)** PLS-DA score plot of control group vs. model group in positive ion mode. **(B)** PLS-DA scores of BOS group vs. model group in positive ion mode. **(C)** Overall PLS-DA scores in positive ion mode. **(D)** PLS-DA scores of control group vs. model group in negative ion mode. **(E)** PLS-DA scores of BOS group vs. model group in negative ion mode. **(F)** Overall PLS-DA scores in negative ion mode.

Partial least squares discriminant analysis (PLS-DA) is a widely used linear classification method known for its exceptional classification capabilities (29). In the field of metabolomics data analysis, PLS-DA remains the most widely used classification approach. This method integrates a regression model with dimensionality reduction techniques and uses a distinct discriminant threshold to perform discriminant analysis on the regression results. The research results demonstrate the successful establishment of the acetic acid-induced GU model, as evidenced by the PLS-DA score (*n* = 6), which clearly separates the control group from the model group into discrete clusters. Furthermore, the BOS group is clearly separated from the model group, indicating the presence of significant differences. Looking at the control, model and BOS groups, the results suggest that BOS has the potential to modulate the atypical metabolic state (Figure 3).

### 3.5 OPLS-DA analysis

**Figure 4:**
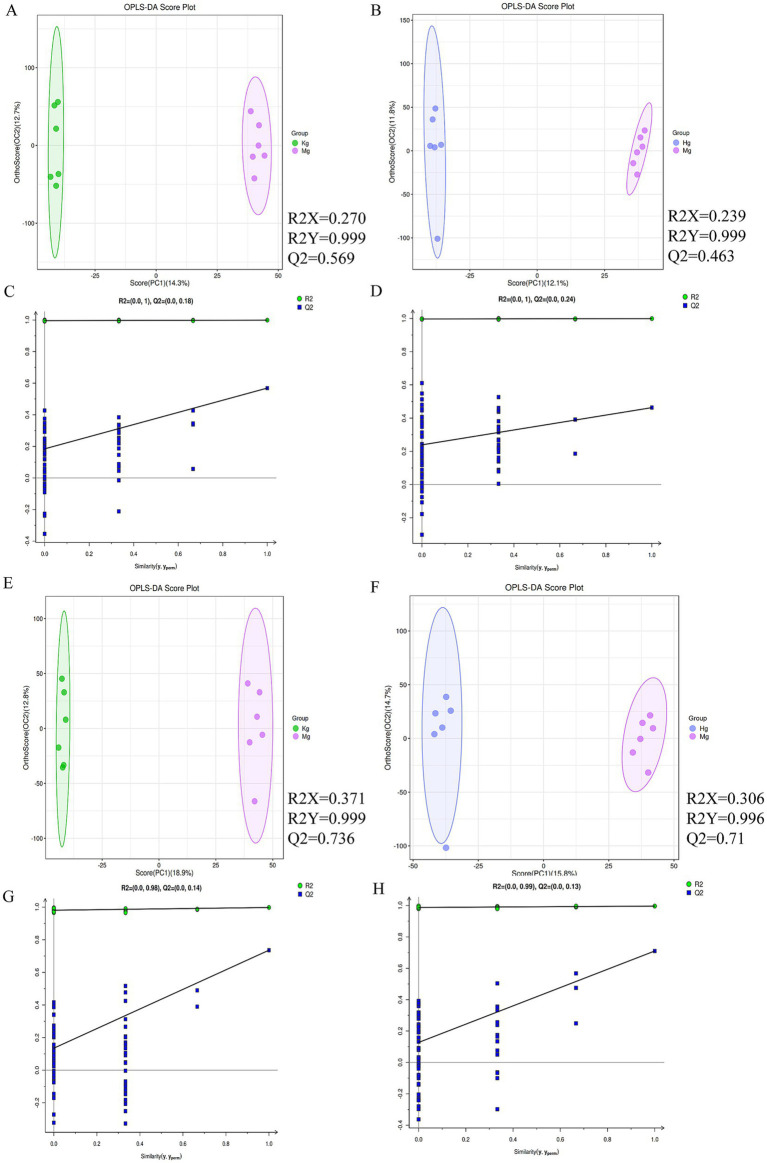
**(A)** OPLS-DA scores between normal and model groups in positive ion mode. **(B)** Cross-validation results between normal and model animal groups in positive ion mode. **(C)** OPLS-DA score map between BOS group and model group in positive ion mode. **(D)** Cross-validation results between animal groups of BOS group and model group in positive ion mode. **(E)** OPLS-DA score map between normal group and model group under negative ion mode. **(F)** Cross-validation results between normal and model animal groups in negative ion mode. **(G)** OPLS-DA scores between BOS group and model group under negative ion mode. **(H)** Cross-validation results between animal groups of BOS group and model group under negative ion mode. *n* = 6 per group.

In order to efficiently analyze the metabolites for filtering and classification purposes, and to eliminate irrelevant noise such as intra-group variation and cross-over factors, the OPLS-DA method was applied to the study of each experimental cohort. Based on the OPLS-DA scores (*n* = 6), all samples fall within the Hotelling *T*^2^ ellipse representing the 95% confidence interval. With the exception of the cumulative *Q*^2^ value observed in the model and high dose groups in the ESI+ mode, the cumulative values of *R*^2^*Y* and *Q*^2^ in the other modes are greater than 0.5. These results underline the reliability of the OPLS model in discriminating the control group from the model and BOS groups. Furthermore, the model demonstrates its effectiveness in identifying disparities between the control, model and BOS groups (Figure 4).

### 3.6 Identification of differential metabolites

**Figure 5:**
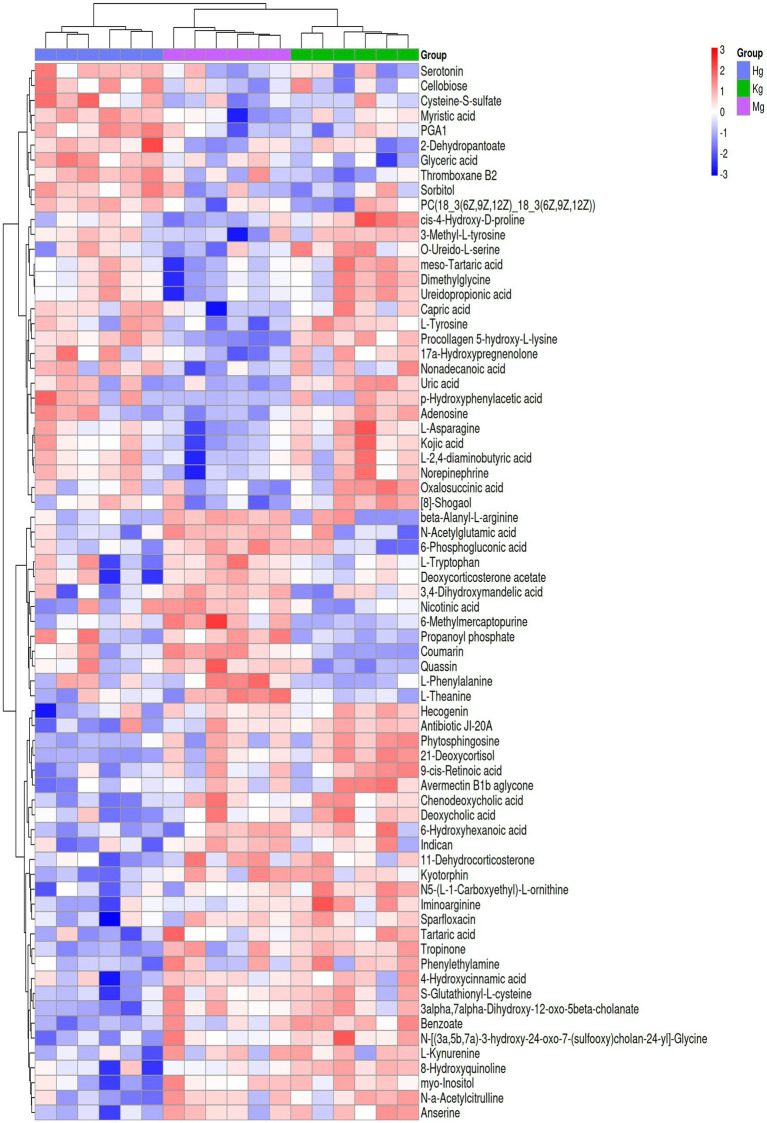
Results of potential identifiers of rat plasma metabolism.

To provide a comprehensive and systematic overview of differential metabolites, potential metabolites identified by both positive and negative ion modes were merged and were associated. A total of 500 signals were detected in the control, model and BOS groups. For clustering and identification purposes, metabolites showing significant contributions were selected for ANOVA analysis based on the VIP and *p*-value thresholds mentioned above. The METLIN and Metaboanalyst databases were then used to identify candidates with significantly altered metabolic biomarkers. As a result, 18 potential biomarkers were identified, including 14 ESI+ patterns and 4 ESI− patterns. Comparison with the control group revealed that the model group showed significant upregulation of six metabolites, namely 6-methylmercaptopurine, beta-alanyl-l-arginine, 3,4-dihydroxymandelic acid, *N*-acetylglutamic acid and coumarin. Conversely, metabolites such as dimethylglycine, 4-diaminobutyric acid, ureidopropionic acid and l-asparagine were significantly down-regulated (see Supplementary Table S1 and Figure 5). These differences in metabolic compounds indicate potential biomarkers that could be used to differentiate between GU and healthy conditions. Furthermore, changes in these compounds may have the potential to predict the onset and progression of GU. After treatment with BOS, a significant down-regulation of six metabolites was observed, including 6-methylmercaptopurine, coumarin, 2,4-dihydroxymandelic acid, *N*-acetylglutamic acid and beta-alanyl-l-arginine. In contrast, other metabolites showed significant increases. This research provides valuable insights into the onset and progression of GU and the identification of potential treatment targets. It also helps to elucidate the molecular mechanisms underlying the therapeutic effects of BOS in the treatment of GU.

### 3.7 Metabolic pathway analysis

**Figure 6:**
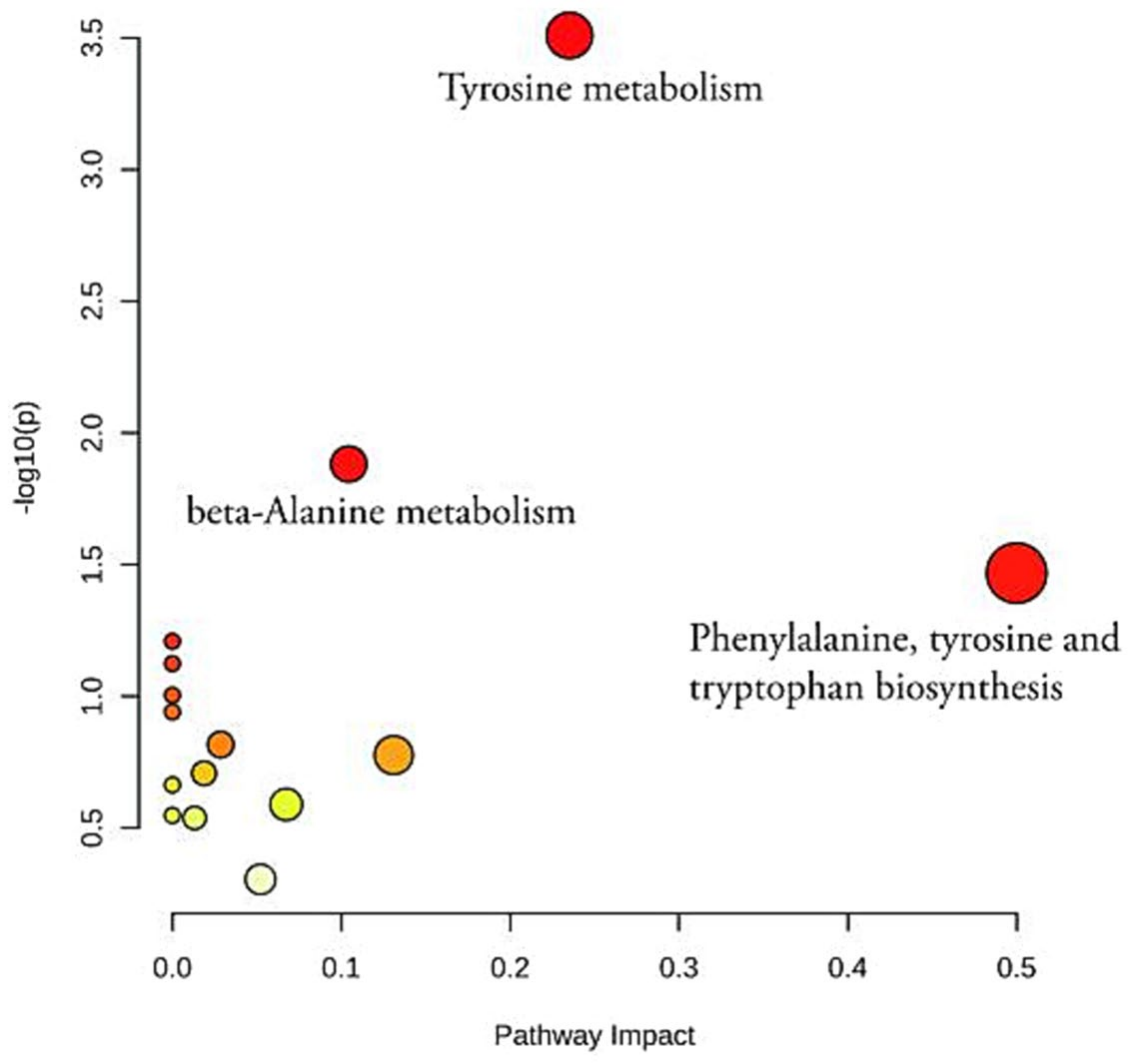
An analysis of KEGG enrichment in positive and negative ion modes of plasma metabolites from rats.

To gain a deeper understanding of the changes in metabolite content, a pathway enrichment analysis was performed on the differential metabolites between the different groups using the KEGG code and Meta PA. The analysis identified three pathways that were significantly altered in the control, model and BOS groups (*p* < 0.05). These pathways include phenylalanine, tyrosine and tryptophan biosynthesis, tyrosine metabolism and beta-alanine metabolism. The overlapping pathways provide insight into the mechanism of acetic acid-induced gastric ulceration in rats and the protective and therapeutic effects of BOS against GU progression. In particular, the pathway involved in the biosynthesis of phenylalanine, tyrosine and tryptophan is closely linked to the development of GU, the role of BOS and the treatment of GU, as shown in Figure 6.

## 4 Discussion

The acetic acid-induced GU rat model has become a key tool in GU research. Numerous studies have consistently shown that acetic acid has the ability to damage the gastric mucosa, triggering an inflammatory cascade that ultimately leads to the formation of GU. In addition, acetic acid has the ability to affect several important metabolic processes, including lipid metabolism, protein metabolism and energy metabolism, which may contribute to gastric mucosal damage and impede the healing process (4, 30). In recent years, researchers have developed innovative experimental designs and analytical methods to improve our understanding of the acetic acid-induced GU rat model. Numerous investigations have shown that natural products have the potential to mitigate damage in the acetic acid-induced GU rat model (6, 20, 31). For example, several studies have documented that BOS and its extracts are able to suppress inflammatory responses and oxidative stress, thereby reducing the risk of developing GU (9). These findings provide new perspectives for the development of novel therapeutic strategies for the treatment of GU.

Using non-targeted metabolomics technology, we investigated the metabolomic characteristics of BOS and its ability to protect against acetic acid-induced GU in rats. The results of the research indicated a significant change in the protective effect of BOS on acetic acid-induced GU in rats, affecting various metabolites such as dimethylglycine, l-2,4-diaminobutyric acid, ureidopropionic acid, l-asparagine, kojic acid, coumarin, meso-tartaric acid. Expression levels of 18 metabolites including hydroxyphenylacetic acid and norepinephrine. Studies suggest that Dimethylglycine may provide protection against GU. It is thought to improve the body’s antioxidant capacity and reduce inflammatory responses, thereby helping to protect the stomach lining from damage (32, 33). Coumarin is a naturally occurring aromatic compound found in many plant foods. It has a number of biological effects, including anti-inflammatory, antioxidant and anti-tumor properties, although it also has some cytotoxic effects. In the human body, coumarin can be metabolized and excreted by multiple pathways, and some of its metabolites may be more cytotoxic (34). Studies have shown that coumarin and its metabolites may damage gastric mucosal cells under certain circumstances, leading to GU (35, 36). In addition, research has suggested a possible link between the metabolite coumarin and *Helicobacter pylori* infection, a major contributor to GU. However, current research is insufficient to establish a direct link between coumarin and the development of GU. In addition, GU is influenced by many other factors and further research is needed to elucidate the role and mechanism of coumarin in its occurrence and development. Kojic acid, a naturally occurring organic compound, exhibits various biological activities including antioxidant, antibacterial and anti-inflammatory properties. It is widely used in areas such as skin care and as a food additive. Several studies suggest that kojic acid may have a protective effect on the stomach lining, potentially alleviating the symptoms of peptic ulcers and aiding in the healing process of the stomach lining. In addition, kojic acid may protect the gastric mucosa by inhibiting the oxidative stress response and reducing the release of inflammatory mediators (37). Some studies show that kojic acid may also inhibit the growth of *Helicobacter pylori*, thereby helping to prevent and treat GU. While the exact link between kojic acid and the development of peptic ulcers is still unclear in current research, some studies suggest that it may offer some protection to the stomach lining and help prevent and treat peptic ulcers. The link between BOS and its ability to protect against GU is suggested by the presence of certain metabolites, particularly amino acids. Certain compounds have the ability to control important metabolic processes, such as the breakdown of amino acids, in order to minimize the damage caused by GU.

The results of KEGG enrichment analysis suggest that BOS could potentially mitigate GU damage by affecting various pathways involved in phenylalanine, tyrosine and tryptophan biosynthesis, as well as beta-alanine metabolism. Phenylalanine is involved in its own degradation and in the production of tyrosine and tryptophan, suggesting that acetic acid-induced GU have a major impact on phenylalanine metabolism. Tryptophan metabolism occurs primarily through the kynurenine, 5-hydroxytryptamine and indole pathways, resulting in the production of bioactive compounds that play a role in controlling various physiological processes such as inflammation, immunity and nerve function. Tyrosine metabolism is about the many ways in which tyrosine is catabolized and metabolized or transformed to produce a variety of biologically important molecules. In particular, tyrosine is metabolized to produce hormones such as thyroxine and triiodothyronine, as well as neurotransmitters such as dopamine (DA) and epinephrine. And in the GI tract, DA binding to receptors can be involved in the regulation of several gastrointestinal functions. For example, DA regulates gastric acid and pepsin secretion and inhibits basal gastric acid secretion Agonist D2 receptors also inhibit histamine and carbachol-induced gastric acid secretion (38, 39). Inhibition of D1 receptors and activation of D2 receptors significantly increase pepsin secretion (40, 41). Second, DA can also affect the gastrointestinal mucosal barrier. DA inhibits gastric and duodenal ulcer formation via D1 receptors, whereas agitation of D2 receptors is pro-ulcerogenic (40). In addition, DA affects epithelial cell ion secretion. DA promotes distal colonic Cl− absorption and HCO_3_− secretion, and this effect is mediated through β1 and β2 adrenergic receptors (42, 43). DA promotes K^+^ secretion from duodenal mucosa via D1 receptor-mediated cyclic adenosine monophosphate (cAMP) pathway (44). DA also affects gastrointestinal motility. DA can inhibit gastrointestinal motility (45–47). Norepinephrine improves the defense of the gastric wall mucosa and significantly accelerates the vasoconstriction of the blood vessels surrounding the bleeding, thus reducing the risk of rebleeding (48). Research shows that tryptophan metabolites are potential therapeutic targets that can modulate disease progression by regulating tryptophan metabolism (49). The experimental results confirmed that after using acetic acid to induce GU, the levels of norepinephrine, *p*-hydroxyphenylacetic acid and l-tyrosine were significantly reduced compared to the control, and the level of 3,4-dihydroxymandelic acid was significantly increased. This unusual change may indicate a disruption in amino acid metabolism. After BOS treatment, there was a significant increase in the model group (*p* < 0.05), while the level of 3,4-dihydroxymandelic acid decreased significantly. The above results suggest that BOS may promote the healing of acetic acid-induced gastric damage by controlling the production of phenylalanine, tyrosine and tryptophan.

While our findings hold significant value, they are not without limitations. Firstly, our focus was solely on elucidating the metabolomic attributes of BOS and its prophylactic effect on acetic acid-induced GU in rats, without delving into a comprehensive mechanistic investigation. To further enhance our understanding, we could embark on a more in-depth exploration of the underlying mechanisms of BOS and its protective role by utilizing gene knockout mouse models, proteomics analysis, and cell culture techniques. Additionally, it is imperative to conduct further research on the protective capabilities of BOS in diverse GU models, as well as assess its translational potential in humans.

## 5 Conclusion

In conclusion, the results of the non-targeted metabolomic analysis suggest a potential protective role of BOS, presumably mediated through the modulation of metabolic pathways and specific metabolites, including phenylalanine, tyrosine, and tryptophan. Future studies are warranted to further investigate the underlying mechanisms of action and explore potential applications.

## Data Availability

The original contributions presented in the study are included in the article/Supplementary material, further inquiries can be directed to the corresponding author.
