# Supplementary material for: Protective effect of Bletilla ochracea Schltr. against acetogenic gastric ulcer in rats based on non-targeted metabolomics
Source: Front Med (Lausanne). 2024 Nov 28;11:1447566. doi: 10.3389/fmed.2024.1447566 (PMC11634584; doi:10.3389/fmed.2024.1447566)
Supplement: Supplementary file 1 [file Table_1.DOCX]

Supplementary Material

# Supplementary and Tables

**Table 1** 18 potential biomarkers identified and trends.

| Name | ID | *m/z* | formula | KEGG | VIP | pos/neg | Changed trend | |
| --- | --- | --- | --- | --- | --- | --- | --- | --- |
|  |  |  |  |  |  |  | Mg/Kg | Hg/Mg |
| Dimethylglycine | M104T37 | 104.0712 | C_4_H_9_NO_2_ | C01026 | 1.9980 | pos | down | up |
| L-2,4-diaminobutyric acid | M118T283 | 118.0652 | C_4_H_10_N_2_O_2_ | C03283 | 1.8252 | pos | down | up |
| Ureidopropionic acid | M133T144 | 133.0609 | C_4_H_8_N_2_O_3_ | C02642 | 1.9900 | pos | down | up |
| L-Asparagine | M133T677 | 133.1007 | C_4_H_8_N_2_O_3_ | C00152 | 1.9134 | pos | down | up |
| Kojic acid | M133T677 | 133.1007 | C_4_H_8_N_2_O_3_ | C00152 | 1.9134 | pos | down | up |
| Coumarin | M147T228 | 147.0438 | C_9_H_6_O_2_ | C05851 | 1.7204 | pos | up | down |
| meso-Tartaric acid | M151T957 | 151.0354 | C_4_H_6_O_6_ | C00552 | 1.8191 | pos | down | up |
| p-Hydroxyphenylacetic acid | M153T33_1 | 153.0911 | C_8_H_8_O_3_ | C00642 | 2.1181 | pos | down | up |
| Norepinephrine | M170T455 | 169.9772 | C_8_H_11_NO_3_ | C00547 | 2.1234 | pos | down | up |
| 3,4-Dihydroxymandelic acid | M184T297 | 184.1696 | C_8_H_8_O_5_ | C05580 | 1.9897 | pos | up | down |
| N-Acetylglutamic acid | M189T146 | 189.0877 | C_7_H_11_NO_5_ | C00624 | 2.3231 | pos | up | down |
| 3-Methyl-L-tyrosine | M195T595 | 195.1019 | C_10_H_13_NO_3_ | C20800 | 1.8411 | pos | down | up |
| beta-Alanyl-L-arginine | M245T266 | 245.1495 | C_9_H_19_N_5_O_3_ | C05340 | 2.2702 | pos | up | down |
| 17a-Hydroxypregnenolone | M315T680 | 315.2305 | C_21_H_32_O_3_ | C05138 | 2.2347 | pos | down | up |
| 6-Methylmercaptopurine | M166T100 | 166.0162 | C_6_H_6_N_4_S | C16614 | 1.9408 | neg | up | down |
| L-Tyrosine | M181T166 | 181.0681 | C_9_H_11_NO_3_ | C00082 | 1.7374 | neg | down | up |
| Procollagen 5-hydroxy-L-lysine | M198T523 | 197.8068 | C_7_H_13_N_3_O_3_R_2_ | C01211 | 2.5695 | neg | down | up |
| 6-Phosphogluconic acid | M257T720 | 257.2111 | C_6_H_13_O_10_P | C00345 | 1.8914 | neg | up | down |
